# Fate of Aflatoxins and Fumonisins During Gluten‐Free Pasta Processing: Untargeted ^13^C‐Labelling LC‐HRMS Based Approach

**DOI:** 10.1002/jms.70106

**Published:** 2026-09-11

**Authors:** Eleonora Rollo, Alexandra Schamann, Maria Doppler, Alexandra Malachová, Rudolf Krska, Michele Suman

**Affiliations:** ^1^ Barilla G. R. F.lli SpA Analytical Food Science Parma Italy; ^2^ Department of Food and Drug University of Parma Parma Italy; ^3^ FFoQSI GmbH—Austrian Competence Centre for Feed and Food Quality, Safety and Innovation Tulln Austria; ^4^ Core Facility Bioactive Molecules: Screening and Analysis BOKU University Tulln Austria; ^5^ Institute of Bioanalytics and Agro‐Metabolomics, Department of Agricultural Sciences BOKU University Tulln Austria; ^6^ Institute for Global Food Security, School of Biological Sciences Queen's University Belfast Belfast UK; ^7^ Department for Sustainable Food Process Catholic University Sacred Heart Milan Italy

**Keywords:** aflatoxin B_1_, degradation products, food processing, fumonisins, gluten‐free pasta

## Abstract

Based on previous experience and current knowledge, it is assumed that mycotoxins contaminating raw materials could be degraded or modified due to exposure to thermal and mechanical conditions during food processing, potentially resulting in degradation and/or conversion products. Nowadays, most of the information on the novel degradation products of mycotoxins is still missing; the identification and characterization of their structure and their toxicity potential is of high interest for risk assessment strategies. In the present study, the fate of aflatoxins and fumonisins during the production of gluten‐free pasta made from maize flour was investigated. Gluten‐free pasta was produced from both spiked dough and naturally contaminated flour, processed under pilot plant conditions that mimic the industrial conditions, resulting in a technologically feasible product. Possible degradation products of the spiked mycotoxins were identified using an untargeted stable isotope labelling liquid chromatography high‐resolution mass spectrometry approach. No degradation product of aflatoxin B_1_ was detected above the detection limit of the experimental setup, and only one possible degradation product of fumonisin B_1_ or B_2_ at very low levels was found. The results may suggest the stability of these mycotoxins under the production conditions of gluten‐free pasta.

## 1 Introduction

Pasta is produced and consumed worldwide, with Italy being the leading producer in the European Union, manufacturing 4.2 million tons in 2023 [1]. Traditionally, pasta is made from durum wheat and thus contains gluten. However, in recent years, there has been a growing trend of people avoiding gluten in their diets due to celiac disease or gluten intolerance either because of fashion or the belief that gluten‐free products are healthier [2]. This shift is reflected in the expanding global market for gluten‐free products, which was valued at $7.70 billion in 2024 and is projected to reach $11.48 billion by 2029 [3]. Gluten‐free pasta is typically made from maize or rice [4], both of which are often contaminated with mycotoxins such as aflatoxins (AFs) and B‐group fumonisins (FBs), making strict quality control essential. Due to the harmful effects of mycotoxins, many countries have implemented regulations to limit exposure. In the European Union, the maximum allowable levels in maize flour are set at 2 μg/kg for AF B_1_ (AFB1) and 1000 μg/kg for the sum of fumonisins B_1_ (FB1) and B_2_ (FB2) [5].

Mycotoxin levels in food are primarily monitored using liquid chromatography–mass spectrometry (LC–MS) [6]. Typically, food samples are tested only for the intact forms of mycotoxins [7]. However, these toxins can undergo degradation or modification due to non‐enzymatic reactions, such as exposure to heat during food processing. As a result, modified, matrix‐associated or masked mycotoxins may form at the expense of their free forms [8, 9, 10, 11]. In these altered forms, mycotoxins often go undetected by routine quality control methods [7]. However, a reduction in mycotoxin levels does not necessarily mean a decrease in toxicological risk. In some cases, degradation products can be even more toxic than the original mycotoxins and may have different toxicokinetic and toxicodynamic properties [12]. Although numerous studies have examined mycotoxin reduction during food processing such as thermal treatment, information on novel degradation products remains scarce [11, 13, 14]. Therefore, identifying and characterizing these compounds, as well as assessing their potential toxicity, is crucial for improving risk assessment strategies [15, 16].

For FB1, thermal reaction products that form in the presence of reducing sugars include *N*‐(carboxymethyl)‐fumonisin B_1_ (NCM‐FB1) and *N*‐(1‐deoxy‐D‐fructos‐1‐yl)‐fumonisin B_1_ (NDF‐FB1) [17]. Matsuo et al. [18] further identified NDF‐FB2 and NDF‐FB3, in addition to NDF‐FB1, in corn samples. In a scientific opinion on the risks associated with fumonisins, the Panel on Contaminants in the Food Chain of the European Food Safety Authority highlighted that these toxins can also covalently bind to macromolecules such as starch and proteins via their reactive tricarboxylic acid side chains [19]. However, direct quantification of these bound fumonisins in food is not feasible, as they must first be released through chemical hydrolysis [20]. Consequently, matrix‐bound fumonisins are typically assessed indirectly by measuring free fumonisins and hydrolyzed fumonisins before and after chemical hydrolysis or following macromolecule digestion [21, 22]. Other modified forms of fumonisins include fatty acid esters of FB1 (O‐fatty acyl FB1, EFB1) and fumonisins with variations in fatty acid chain length and esterification positions (3‐O‐, 5‐O‐ or 10‐O‐acyl fumonisins) [19]. Although numerous degradation pathways have been described in the literature for AFs using various physical techniques, there is still very little research available on the formation of modified AFs due to industrial processing.

This study aims to investigate the fate of AFB1 and FBs during the production of gluten‐free pasta made from maize flour in a pilot plant experiment that replicates industrial processing to obtain a technologically feasible product. To identify all possible degradation or conversion products of the mycotoxins, an untargeted stable isotope labelling (SIL) liquid chromatography–high‐resolution mass spectrometry (LC‐HRMS) approach, which leverages the use of stable isotopes occurring naturally at low abundance [23], was followed. This method has been successfully applied in a previous study to track the fate of deoxynivalenol during the production of crackers, biscuits and bread [12, 24].

## 2 Materials and Methods

### 2.1 Chemicals and Reagents

HiPerSolv Chromanorm HPLC gradient grade acetonitrile was obtained from VWR International GmbH (Vienna, Austria), acetic acid (LC–MS gradient grade) and LiChropur ammonium acetate from Sigma Aldrich (Vienna, Austria), LC–MS Chromasolv methanol from Fisher Scientific (Illkirch, France) and NaCl from Merck KGaA (Darmstadt, Germany). Ultra‐pure water purified in‐house by a Purelab Ultra system (ELGA LabWater, Celle, Germany) was used. For the spiking of maize flour, the AF standards AFB_1_ (2.0 μg/mL) in acetonitrile and U‐[^13^C_17_]‐AFB_1_ (0.5 μg/mL) in acetonitrile (both Biopure, Romer Labs, Tulln, Austria) and the fumonisin standards Biopure Mix 3 (fumonisin B_1_ and B_2_ in acetonitrile/water, Romer Labs, Tulln, Austria) and LIB′UP U‐[^13^C_34_]‐fumonisins B_1_ and B_2_ (each 10.0 μg/mL) mixture in acetonitrile/water (Libios, Vindry Sur Turdine, France) were used.

### 2.2 Samples

Compliant maize flour, sourced from an Italian supplier, was used for the spiking experiments. Naturally contaminated maize kernels, harvested from a highly contaminated field during a previous harvesting campaign, were used for the production of gluten‐free maize flour at the pilot plant scale.

### 2.3 Untargeted Tracer Fate Study of AFB1 and FB1 and FB2

An untargeted metabolomics workflow, which was first used to detect metabolites of deoxynivalenol in wheat [25], was used to study the fate of AFB1 as well as FB1 and FB2 during pasta making.

- To assess the degradation of AFB1 during lasagne production, 20 g of compliant maize flour was spiked with either 65 μg/kg AFB1 (non‐labelled) or 65 μg/kg AFB1, plus an equivalent amount of U‐[^13^C_17_]‐AFB1.
- For the analysis of the degradation of fumonisins, 20 g of compliant maize flour was spiked with either 400 μg/kg FB1 and FB2 (non‐labelled) or 400 μg/kg FB1 and FB2, plus an equivalent amount of U‐[^13^C_34_]‐FB1 and U‐[^13^C_34_]‐FB2.

After pipetting the mycotoxin standards onto the flour, the bowls were sealed with a porous parafilm and left under the fume hood for approximately 60 min to allow solvent evaporation before proceeding with lasagne production. In addition to the spiked maize flour, lasagne was also made from 20 g compliant non‐spiked maize flour for comparison.

**FIGURE 1:**
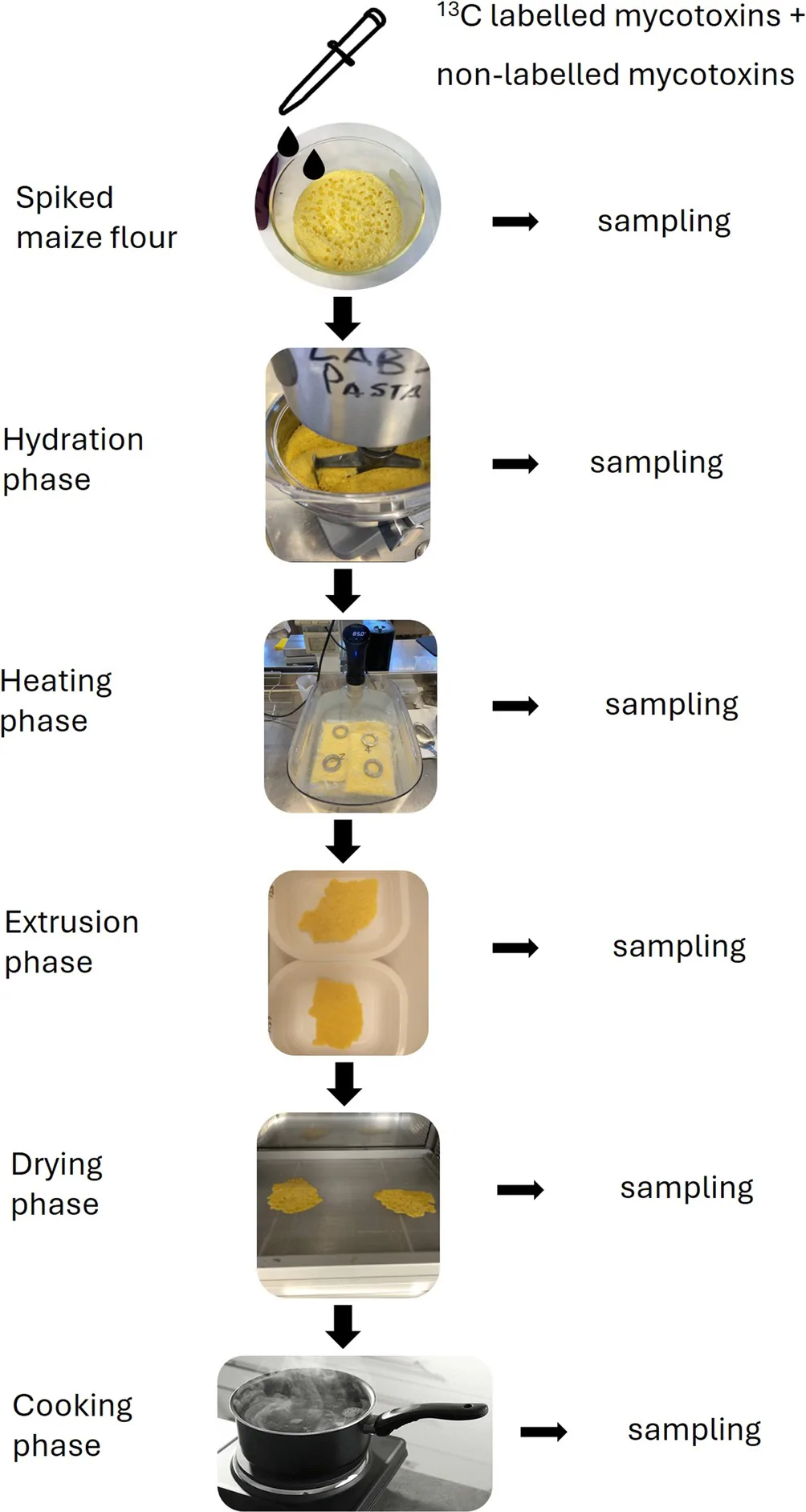
Workflow of gluten‐free pasta production and sampling (4 g) for subsequent analysis.

The workflow of the gluten‐free pasta production is illustrated in Figure 1. To achieve the desired initial moisture content for the lasagne production, 65% water was added to 20 g spiked or non‐spiked maize flour in a small bowl and mixed by stirring. Then the dough was transferred into vacuum‐sealed bags and heated in a thermostatic bath at 85°C for 12 min, ensuring the dough temperature reaching 75°C (Figure 1). Next, the dough was rolled out manually using a rolling pin to form a thin lasagne sheet. The sheet was then placed in a drying chamber, where a drying cycle (360 min) was applied, regulating humidity, air speed, time and temperature (max. 80°C; Italpast, Parma, Italy). The water content of the dried pasta was checked (Sartorius, Göttingen, Germany). After drying, the pasta was cooked for 7 min in 200 mL of boiling water containing 7 g/L NaCl. Samples (approximately 4 g each) were collected at multiple stages of production for subsequent analysis.

#### 2.3.1 Extraction and Sample Preparation

The samples ‘dried pasta’ and ‘cooked pasta’ were homogenized and ground for a few seconds using a blender (Osterizer). This step was not required for the remaining samples, as these were already sufficiently fine for extraction. For each sample, 0.75 g was weighed in duplicate (two technical replicates). Mycotoxins were extracted using 3 mL of extraction solvent (acetonitrile/water/acetic acid, 79/20/1, v/v/v) by shaking the samples horizontally for 90 min on a rotary shaker (GFL 3017, Burgwedel, Germany). After centrifugation at 4000 rpm for 10 min (Sigma 3‐18 KS, Linder Labortechnik, Vienna, Austria), 0.5 mL of the supernatant was transferred into deactivated amber glass HPLC vials (Waters, Vienna, Austria), and an equal volume (0.5 mL) of acetonitrile/water/acetic acid (20/79/1, v/v/v) was added. Then, the samples were vortexed and analysed by LC‐HRMS and the AFB1 samples additionally by LC–MS/MS (see also Section 3.1.2). In addition, the spiking solutions of the non‐labelled standards (AFB1 at 0.1 μg/mL; FB1 and FB2 at 0.5 μg/mL) and the mixture of non‐labelled and ^13^C‐labelled standards (at the same respective concentrations) were analysed by LC‐HRMS.

#### 2.3.2 LC‐HRMS Measurement

The sample extracts were analysed in a randomized order on an UHPLC system coupled to an Orbitrap Q Exactive HF mass spectrometer (Thermo Scientific, Bremen, Germany) applying the method described in detail by Doppler et al. [26]. Chromatographic separation was achieved on a RP C18 column (3.5 μm; 2.1 × 150 mm; XBridge; Waters; Milford; Massachusetts; USA) using a 20‐minute gradient method. The temperature in the autosampler was set to 10°C and in the column oven to 25°C. For each sample, a volume of 2 μL was injected. Full‐scan mass spectra were recorded from *m/z* 100 to 1000 in fast polarity switching mode and with a resolution of 120 000 (FWHM at *m/z* 200). Quality control standards were injected regularly throughout the whole sequence.

#### 2.3.3 Automated Data Processing

The software MetExtract II was used for LC‐HRMS data evaluation, which is described in detail by Bueschl et al. [27]. The datasets corresponding to the AF‐ and fumonisin‐spiked samples were analysed separately. Briefly, data‐processing was performed as follows: Full‐scan mass spectrometer data were checked for characteristic isotopologue pairs of mass peaks of *M*, which represents a possible degradation or transformation product derived from the unlabelled standard, and *M′*, which represents the corresponding product derived from the uniformly ^13^C‐labelled standard (Table 1). These pairs have a mass difference (Δ*m*/*z*), which corresponds to the number of tracer‐derived ^13^C atoms incorporated in *M'* (Xn). Further, co‐elution of *M* and *M′* as well as the conformity of their concentrations, as labelled and non‐labelled standards were spiked at the same concentrations, were verified. To eliminate potential false positives, peaks detected in control samples (without spiked mycotoxins) were subtracted from those in spiked samples. This ensured that only signals specifically associated with mycotoxin transformation or degradation products were considered for further analysis.

**TABLE 1:** Parameter settings for data processing using MetExtract II.

	Parameter	AFB1	FB1/FB2
	Module	TracExtract	TracExtract
Single file processing	Isotopic enrichment % (N/L)	98.9/99.0	98.9/97.8
	Atom count	17	34
	Δ *m*/*z*	1.00335	
	Min–max Cn atoms	10–17; 34	6–34; 65–69
	Scan range start–stop (min)	1–13	
	Intensity threshold (≥)	10 000	1000
	Intensity cutoff (≤)	0	
	Consider isotopologue abundance	No	
	Mass deviation (±ppm)	6	
	Isotopologs verified (N/L)	2/2	
	Max. isotopologue ratio error % (N/L)	15/15	30/30
	Clustering (ppm)	8	
	Min. number of spectra	3	
	EIC width (±ppm)	5	
	Peak width (min/max scans)	3/11	
	Peak matching (scans)	0	
	Min. peak correlation	0.85	
	Min. convolution correlation	0.85	
Bracketing	Max. *m*/*z* width (±ppm)	12	
	Max. time window (min)	0.1	
	Integration max. time difference (min)	0.1	

#### 2.3.4 LC–MS/MS Measurement

Sample extracts were additionally analysed by LC–MS/MS to quantify mycotoxins. LC–MS/MS measurement was performed as described by Sulyok et al. [28]. Briefly, metabolite analysis of the extracts was performed on a 1290 Series HPLC System (Agilent, Waldbronn, Germany) connected to a QTrap 5500 LC–MS/MS System (Applied Biosystems SCIEX, Framingham, Massachusetts, USA) equipped with Turbo Ion Spray electrospray ionization source. The injection volume was 5 μL. The chromatographic separation was achieved on a Gemini C_18_‐column (150 × 4.6 mm i.d., 5 μm), connected with a C_18_ 4 × 3 mm i.d. security guard cartridge (Phenomenex, Torrance, California, USA) using methanol/water/acetic acid 10:89:1 (v/v/v) and 97:2:1 (v/v/v), both with 5‐mM ammonium acetate, as Eluents A and B at a flow rate of 1000 μL/min. After 2 min at 100% Eluent A, Eluent B was linearly increased to 50% within 3 min. Then, Eluent B was further linearly increased to 100% within 9 min, followed by 4 min at 100% Eluent B and 2.5 min for column equilibration at 100% Eluent A. Results were corrected for apparent recoveries and the humidity levels of the sample taken at the different production steps (e.g., raw material and dough).

### 2.4 Verification of the Presence of Degradation Products in Pasta Made From Naturally Contaminated Maize Flour

**FIGURE 2:**
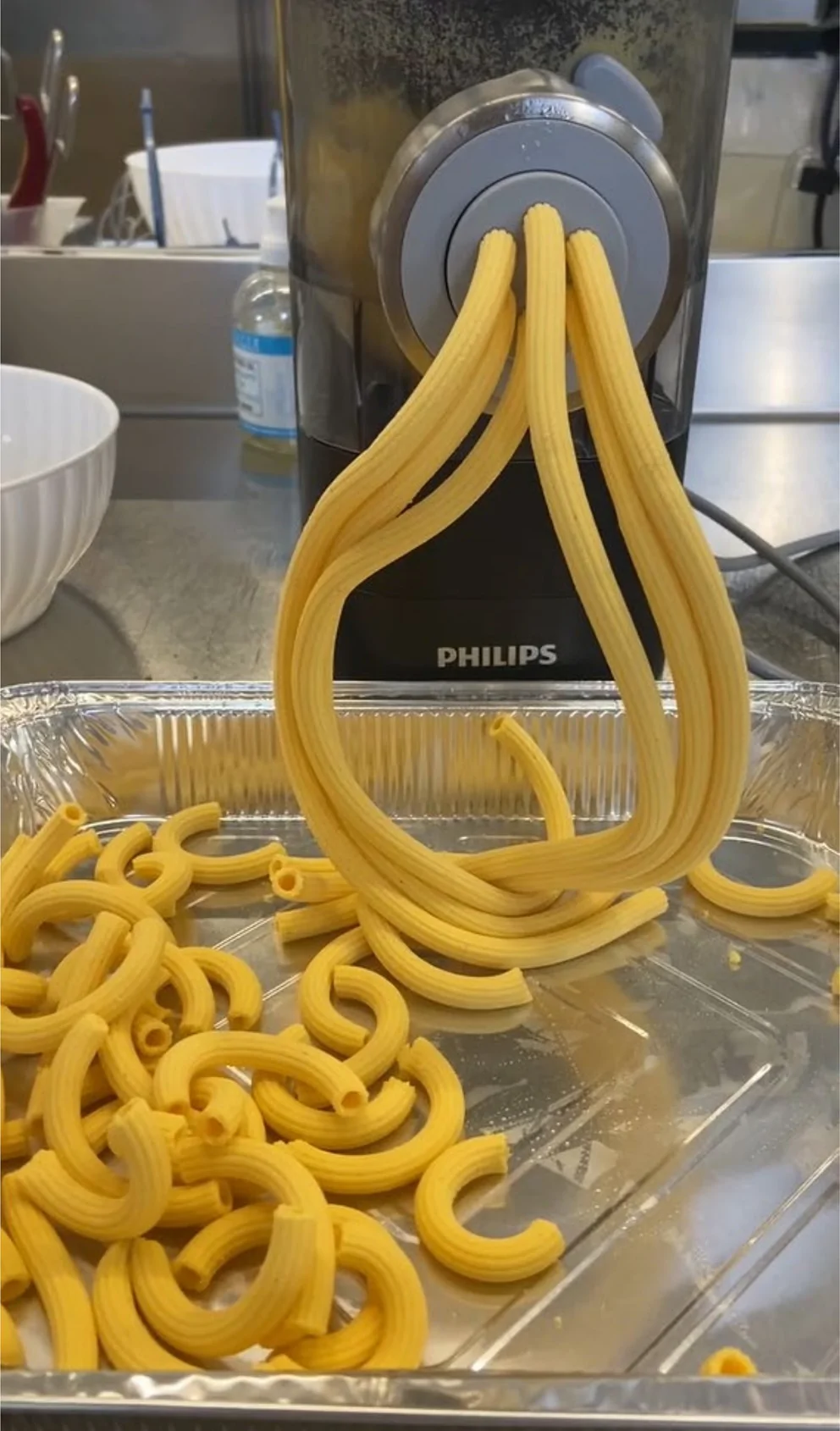
Extrusion phase with the extruder machine equipped with a die for *maccheroni*.

To determine whether the degradation products detected in samples from the spiked lasagne were also present in pasta made from naturally contaminated maize flour, the pasta production process was repeated under comparable conditions as described in Section 2.3, but with an increased maize flour quantity (200 g). The heated dough was then divided into two equal portions: One was used to produce lasagne sheets as described above while the other was used to produce *maccheroni* (Italian shape of pasta, referred to as macaroni in English). For the *maccheroni* production, the dough was extruded using an extruder machine (Philips Pasta Maker, Amsterdam, the Netherlands) equipped with a die for *maccheroni* (Figure 2). Extracts of samples collected at the processing steps outlined in Section 2.3 were analysed as a single replicate using LC‐HRMS.

## 3 Results

### 3.1 Untargeted Tracer Fate Study of AFB1 and FB1 and FB2

#### 3.1.1 Control Experiment

The first step was the production of gluten‐free pasta with compliant maize to optimize the production process (hydration, heating, extrusion, drying and cooking). A key challenge was to minimize raw material amount (20 g) while ensuring a technologically feasible and marketable product. This approach aimed to limit the amount of costly ^13^C‐labelled standard required.

The humidity of the dried lasagne sheets (*n* = 4) was 10.8%, which complies with the legal limit of less than 12.5%. Naturally occurring mycotoxin levels of the compliant maize flour were 1.95 μg/kg AFB1, 625 μg/kg FB1 and 148 μg/kg FB2.

#### 3.1.2 Fate of AFB1

Lasagne sheets were produced from maize flour spiked with either non‐labelled AFB1 or a combination of non‐labelled and uniformly ^13^C‐labelled AFB1, and samples taken at five different production steps (listed in Section 2.3) were analysed.

To enable the identification of potential degradation or conversion products, the SIL approach was applied, which is based on spiking the raw material with both non‐labelled and uniformly ^13^C‐labelled forms of the target mycotoxin standards. While the native (non‐labelled) mycotoxin standard reflects the natural isotopic distribution of carbon (98.93% ^12^C and 1.07% ^13^C), the uniformly labelled standard predominantly contains ^13^C (typically 95%–99% and 1%–5% ^12^C, depending on purity). In HRMS, the native mycotoxin standard primarily produces a signal for its monoisotopic ^12^C isotopologue (*M*) alongside minor signals corresponding to naturally occurring ^13^C isotopologues (*M* + 1, *M* + 2). In contrast, the uniformly labelled standard predominantly produces a signal for the fully ^13^C‐labelled isotopologue (*M′*), with additional minor signals for partially back‐exchanged isotopologues (*M′* − 1, *M′* − 2), which contain one or two ^12^C atoms.

Like mycotoxin standards, putative degradation products formed during pasta production also exhibit these isotope patterns. These patterns were used by the software MetExtract II to filter possible degradation or conversion products from the mass spectrum of toxin extracts of all these samples [27]. No such products of AFB1 were detected.

**FIGURE 3:**
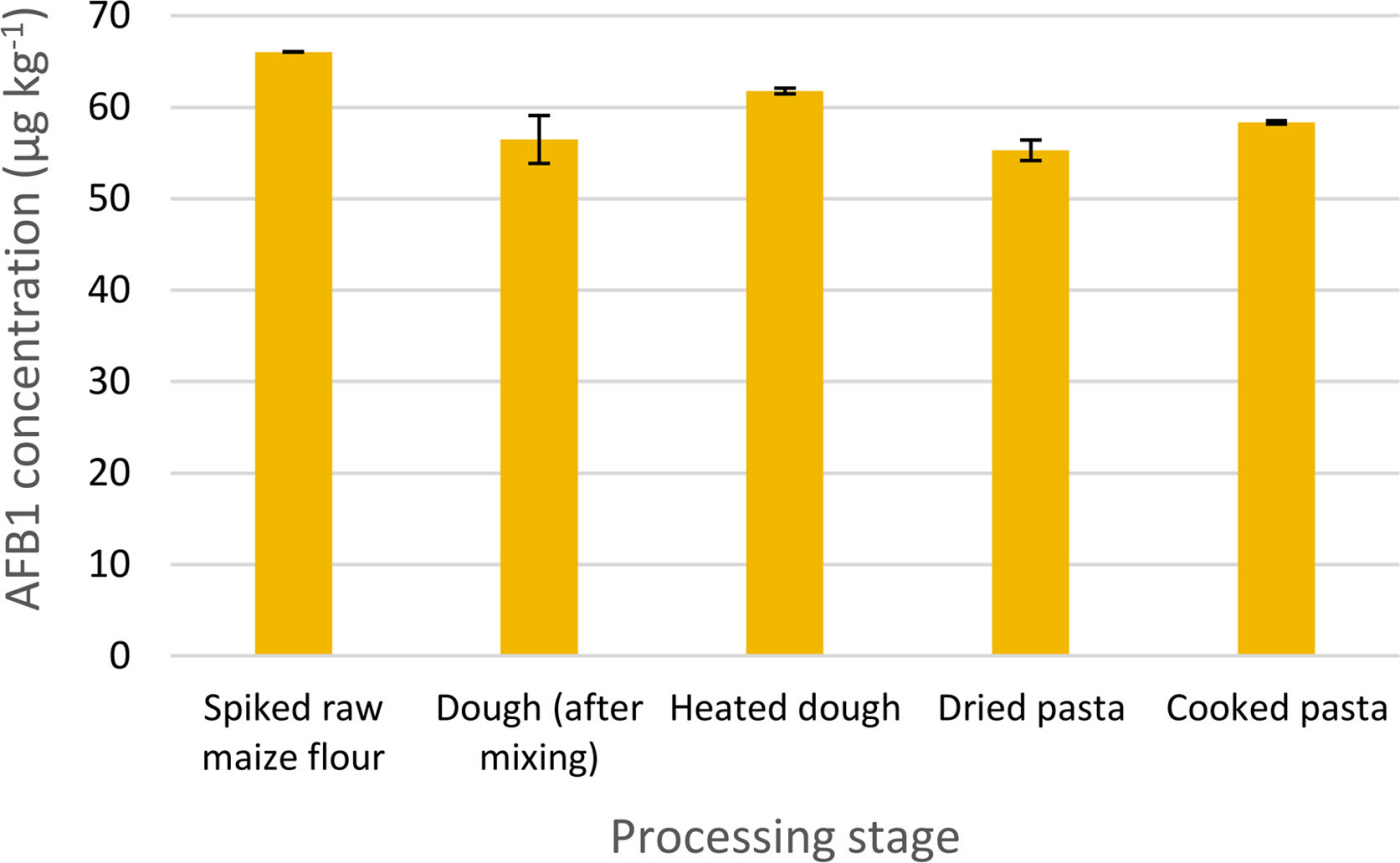
Aflatoxin B_1_ (AFB1) concentrations (μg kg^−1^) measured by LC–MS/MS in extracts of samples collected at different processing stages during lasagne production from maize flour spiked with non‐labelled AFB1 (65 μg kg^−1^). Error bars represent standard deviations of technical replicates (*n* = 2).

To assess the stability of AFB1 during the lasagne production, the mycotoxin was additionally quantified in the sample extracts by LC–MS/MS. As shown in Figure 3, AFB1 levels varied only slightly across samples taken at different production stages. The remaining variations can be attributed to differences in matrix effects, moisture content of the various materials, such as dough, dried or cooked pasta, and the small sample volumes analysed.

#### 3.1.3 Fate of FB1 and FB2

**FIGURE 4:**
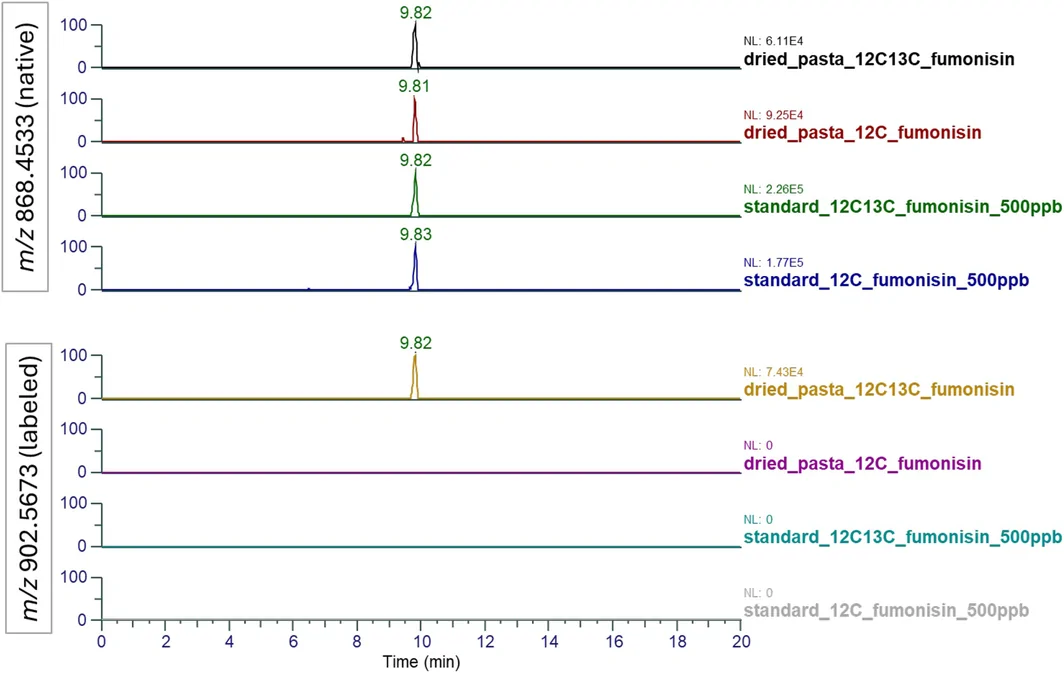
Extracted ion chromatograms of a fumonisin‐derived metabolite in its non‐labelled (*m*/*z* 868.4533) and labelled (*m*/*z* 902.5673) form. Chromatograms are shown for representative dried pasta samples produced from maize spiked with non‐labelled (^12^C) or both, non‐labelled and ^13^C‐labelled fumonisin standard (^12^C^13^C), as well as for the corresponding standards.

**FIGURE 5:**
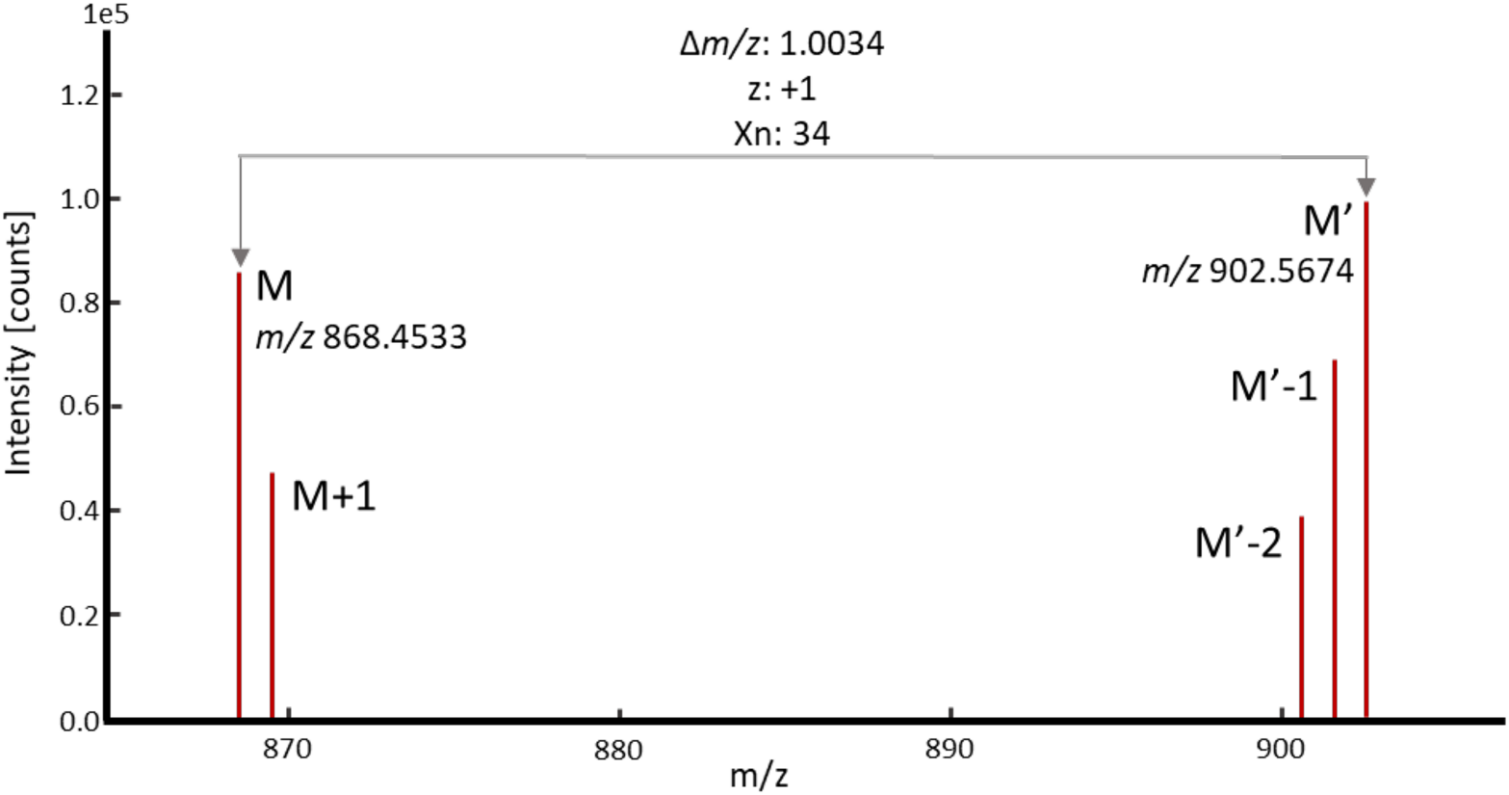
Illustration of the HRMS mass spectrum (positive mode) showing the isotopic pattern of a putative fumonisin‐derived metabolite (*M*, *m/z* 868.4533) detected in an extract of dried pasta produced from maize flour spiked with both fully ^13^C‐labelled and non‐labelled fumonisin B_1_ and B_2_ standards. The left side of the spectrum shows the monoisotopic ^12^C isotopologue (*M*) of the non‐labelled standard together with its *M* + 1 isotopic peak, corresponding to the incorporation of a single ^13^C atom, while the expected *M* + 2 signal is not observed. On the right side, the corresponding fully ^13^C‐labelled isotopologue (*M'*) is detected, accompanied by two partially labelled isotopologues (*M′* − 2 and *M′* − 1).

Lasagne sheets were also produced using maize flour spiked with non‐labelled FB1 and FB2, as well as maize flour spiked with both non‐labelled and ^13^C‐labelled FB1 and FB2. Again, samples were collected at five production steps to monitor potential degradation or conversion products. Initially, three fumonisin‐derived compounds were detected (Table 2). However, two of these metabolites (1 and 2), both containing 28 of the original 34 carbon atoms of spiked fumonisins, were also found in the labelled and the non‐labelled standards as well as in the raw maize flour. This suggests that these two metabolites were most likely contaminants present in both standards and the raw maize flour rather than conversion/degradation products. Accordingly, the signal intensities of Metabolite 2 in the individual samples were similar to those observed in the raw material, remaining below the limit of quantification. In contrast, Metabolite 1 showed slightly higher signal intensities in the dried pasta samples compared with the raw material. However, reliable quantification was not possible. In line with the objective of focusing exclusively on metabolites absent from both the raw material and the standards, these two metabolites will not be discussed in further details in this paper. The third metabolite was present as a contaminant in the non‐labelled standard but was absent in the labelled standard and raw maize flour. At very low intensities, the metabolite was detected in both replicates of the dried pasta samples and in one replicate of the cooked samples, indicating formation from the labelled standard during processing. For illustration, Figure 4 shows extracted ion chromatograms of this metabolite across representative samples. The signal at *m/z* 902.5673 (corresponding to the ^13^C‐labelled form) is present in a dried pasta replicate prepared from maize spiked with labelled and non‐labelled standards, whereas no corresponding signal is observed in samples spiked only with non‐labelled standard or in the pure standards. In contrast, the signal at *m/z* 868.4533 (non‐labelled form) is detected in the non‐labelled standard, the standard mixture and in dried pasta samples. Together, these chromatograms provide evidence supporting that the metabolite retaining all 34 carbon atoms of fumonisin is formed during pasta production from the labelled precursor. However, as shown in Figure 5, the isotope pattern analysis of one sample containing the metabolite in both forms revealed an atypical picture. On the non‐labelled side, only the first isotopologue (*M* + 1), containing a single ^13^C atom, along with the monoisotopic ^12^C isotopologue (*M*) was detected. On the labelled side, two isotopologues (*M' −* 2 and *M' −* 1), incorporating one or two ^13^C atoms, were detected in addition to the monoisotopic ^13^C isotopologue (*M'*). Optimally, the isotope pattern should display a mirror‐image pattern with two isotopologues (*M* + 1 and *M* + 2, *M' −* 2 and *M' −* 1) of the monoisotopic ^12^C or ^13^C isotopologues (*M*, *M′*). Therefore, the metabolite did not meet the quality criteria required for confirmation as a relevant fumonisin‐derived compound.

**TABLE 2:** List of fumonisin‐derived metabolites detected in pasta samples spiked with fumonisin standards using the software MetExtract II.

Metabolite number	*m/z* (*M*)	*m/z* (*M′*)	Δ*m/z*	Xn	Retention time [min]	Detected in non‐labelled fumonisin standard	Detected in ^13^C‐labelled fumonisin standard
1	564.3746 [M + H]^+^	592.4685 [M + H]^+^	28.09394	28	8.97	Yes	Yes
2	548.3795	576.4734	28.09394	28	9.70	Yes	Yes
3	868.4533	902.5674	34.11406	34	9.82	Yes	No

*Note:* Metabolites are listed with their *m/z (M*) (monoisotopic ^12^C isotopologue), *m/z (M′*) (monoisotopic ^13^C isotopologue), Δ*m/z* (mass difference between monoisotopic ^12^C and ^13^C isotopologues) and retention time. The measurements were performed in positive ionization mode using HRMS.

### 3.2 Control of the Formation of Fumonisin‐Derived Metabolites in Pasta Produced Using Naturally Contaminated Maize Flour

Lasagne sheets and *maccheroni* were produced from naturally contaminated maize. The mycotoxin levels in the contaminated maize determined by LC–MS/MS were as follows: AFB1, 74.9 μg/kg; FB1, 708 μg/kg; and FB2, 146 μg/kg. Samples of the maize flour and five further pasta production steps were controlled for the above‐mentioned degradation or conversion products. Metabolites 1 and 2 of the FBs were detected in the naturally contaminated raw maize flour as well as in all the following production steps. The third metabolite was only detected in the dried *maccheroni* sample at very low intensity but not in the raw maize flour, the dried lasagne sample or any other sample of the pasta production.

## 4 Discussion

In the current study, pasta was produced from maize flour spiked with the mycotoxins AFB1 or FB1 and FB2. The aim was to detect degradation or conversion products of the spiked mycotoxin standards in the samples taken after each production step.

The spiking level of AFB1 (65 μg/kg) was significantly higher than the legal limit of 2 μg/kg in maize in the EU [5]. No degradation products of AFB1 were detected above the detection limit of the methodological setup used, suggesting that AFB1 remains mostly stable during the production of gluten‐free pasta under the specified conditions, which included heating at 75°C, drying at a maximum of 80°C and boiling for 7 min. This assumption was further supported by the relatively consistent AFB1 levels observed in samples across the different production steps. The variations seen in Figure 3 should not be overinterpreted, as the study was designed primarily for qualitative analysis rather than quantitative measurement. To prevent the potential formation of degradation products due to an additional drying step, which is not part of the pasta production process, samples were not dried before extraction. Instead, the results were adjusted based on the average humidity levels of each sample type (e.g., dough and dried pasta). However, variations in water content and matrix effects of the different sample types likely still influenced the results. Additionally, the sample volume taken at each production step was very small due to the high cost of ^13^C‐labelled mycotoxin standards. Consequently, the quality of the measured data was affected. We cannot rule out the possibility that degradation products were formed at concentrations below their respective detection limits less than 1% of the spiked AFB1 level.

The results of this study are aligned with the observation that AFs generally exhibit high stability under various conditions [29]. Stoloff and Trucksess [30] demonstrated that the degradation of AFB1 is strongly dependent on the processing conditions of contaminated maize. Experiments employing more challenging thermal production conditions (e.g., higher temperatures or longer treatment times) than in the current experiment resulted in losses of AFB1. For example, boiling maize grit for 30 min resulted in a recovery of 72% ± 9% of the original AFB1, while baking maize flour at 218°C for 20 to 25 min into muffins resulted in an AFB1 recovery of 87% ± 4%. However, these publications did not investigate the formation of degradation products of AFB1.

Only one putative fumonisin‐derived product absent in the raw material was detected above the limit of detection in the current study, suggesting also a relatively high stability of the spiked fumonisins under the tested conditions for the production of gluten‐free pasta. In general, FBs are considered to be quite heat stable and survive thus many food production processes [13]. The stability seems to be significantly influenced by the type of processing condition, as baking maize muffins spiked or naturally contaminated with fumonisins at 204°C for 20 min did not significantly reduce mycotoxin levels, while roasting (dry heating) of maize meal samples at 218°C for 15 min reduced fumonisins nearly completely [31].

The one fumonisin‐derived product (*M*, *m/z* 868.4533) detected in the spiked fumonisin samples was also detected in the dried *maccheroni* sample made from naturally contaminated maize at very low intensities. Thus, based on the results gained, it can be assumed that this unknown metabolite is formed during the drying step of the pasta production. However, it was not detected in all dried pasta samples. Thus, either it is not constantly produced or its concentration in the other dried pasta samples was simply too low that it could have been detected. As the isotope pattern of the metabolite did not fulfil the quality criteria and as the concentration was very low, we did not further focus on this metabolite.

## 5 Conclusion

The fate of AF (AFB1) and fumonisins (FB1 and FB2) was investigated during the production of gluten‐free pasta. The pasta was made from maize flour that was either spiked with these mycotoxins or naturally contaminated, and all processing steps were conducted under pilot plant conditions. Under these conditions, no degradation or conversion products of AFB1 were detected above the detection limit of the analytical method used in this study, suggesting high stability during the production of gluten‐free pasta. For FB1 and FB2, a metabolite (*m/z* 868.4533) was detected at very low intensity. However, due to an insufficient isotope pattern in the mass spectrum, which did not meet our quality criteria, it could not be confirmed as a degradation or conversion product of the spiked fumonisins. Thus, also no confirmed degradation products of FB1 and FB2 were identified within the sensitivity limits of the present experimental setup. These findings should be interpreted in the context of the applied methodology and do not exclude the formation of transformation products below the detection limits or outside the analytical scope of this study.

## Funding

This work has received funding from the European Union’s Horizon Europe Research and Innovation Programme under grant agreement no. 101060698 (FoodSafeR); and the NextGenerationEU—Piano Nazionale Resistenza e Resilienza (PNRR)—Missione 4 Componente 2 Investimento 1.3—Avviso N. 341 del 15 Marzo 2022 del Ministero dell' Università e della Ricerca; Codice progetto PE00000003, Decreto Direttoriale MUR n. 1550 dell' 11 Ottobre 2022 di concessione del finanziamento, CUP D93C22000890001, titolo progetto ‘ON Foods—Research and innovation network on food and nutrition Sustainability, Safety and Security—Working ON Foods’, CUP D93C22000890001.

## Data Availability

The data that support the findings of this study are available from the corresponding author upon reasonable request.
